# A web-based clinical decision tool to support treatment decision-making in psychiatry: a pilot focus group study with clinicians, patients and carers

**DOI:** 10.1186/s12888-017-1406-z

**Published:** 2017-07-21

**Authors:** Catherine Henshall, Lisa Marzano, Katharine Smith, Mary-Jane Attenburrow, Stephen Puntis, Jakov Zlodre, Kathleen Kelly, Matthew R Broome, Susan Shaw, Alvaro Barrera, Andrew Molodynski, Alastair Reid, John R Geddes, Andrea Cipriani

**Affiliations:** 1 OxINMAHR, Faculty of Health and Life Sciences, Oxford Brookes University, Oxford, UK; 2 Oxford Health NHS Foundation Trust, Warneford Hospital, Oxford, UK; 3 Department of Psychology, Middlesex University, London, UK; 4 Department of Psychiatry, University of Oxford, Warneford Hospital, Oxford, OX3 7JX UK

**Keywords:** Focus group, Treatment algorithm, Evidence based decision tool, Decision making

## Abstract

**Background:**

Treatment decision tools have been developed in many fields of medicine, including psychiatry, however benefits for patients have not been sustained once the support is withdrawn. We have developed a web-based computerised clinical decision support tool (CDST), which can provide patients and clinicians with continuous, up-to-date, personalised information about the efficacy and tolerability of competing interventions. To test the feasibility and acceptability of the CDST we conducted a focus group study, aimed to explore the views of clinicians, patients and carers.

**Methods:**

The CDST was developed in Oxford. To tailor treatments at an individual level, the CDST combines the best available evidence from the scientific literature with patient preferences and values, and with patient medical profile to generate personalised clinical recommendations. We conducted three focus groups comprising of three different participant types: consultant psychiatrists, participants with a mental health diagnosis and/or experience of caring for someone with a mental health diagnosis, and primary care practitioners and nurses. Each 1-h focus group started with a short visual demonstration of the CDST. To standardise the discussion during the focus groups, we used the same topic guide that covered themes relating to the acceptability and usability of the CDST. Focus groups were recorded and any identifying participant details were anonymised. Data were analysed thematically and managed using the Framework method and the constant comparative method.

**Results:**

The focus groups took place in Oxford between October 2016 and January 2017. Overall 31 participants attended (12 consultants, 11 primary care practitioners and 8 patients or carers). The main themes that emerged related to CDST applications in clinical practice, communication, conflicting priorities, record keeping and data management. CDST was considered a useful clinical decision support, with recognised value in promoting clinician-patient collaboration and contributing to the development of personalised medicine. One major benefit of the CDST was perceived to be the open discussion about the possible side-effects of medications. Participants from all the three groups, however, universally commented that the terminology and language presented on the CDST were too medicalised, potentially leading to ethical issues around consent to treatment.

**Conclusions:**

The CDST can improve communication pathways between patients, carers and clinicians, identifying care priorities and providing an up-to-date platform for implementing evidence-based practice, with regard to prescribing practices.

## Background

The efficacy of current pharmacological and non-pharmacological interventions in psychiatry is well established and worthwhile, but still far from optimal [1]. Despite the enormous burden of psychiatric disorders worldwide, progress in developing new treatments is slow, in part due to inadequate knowledge of the bio-psychosocial mechanisms underlying mental disorders [2]. To improve clinical outcomes of patients it is important to make use of all available scientific information. Data from existing clinical studies are ideal because they provide better estimates of comparative efficacy between interventions, allowing treatment indications to be personalised, by stratifying results for specific subgroups of patients according to baseline clinical and demographic characteristics [3]. Through performing sophisticated re-analyses of existing datasets, researchers can predict the probability of a treatment response or determine the chances that a person will have a particular side effect [4]. By matching patients with treatments that are more likely to be effective and cause fewer side effects, clinicians can use this information to customize treatments to patients’ needs, thus improving their outcomes. This approach, known as “personalised” or “precision medicine” is now widely used in many fields of medicine [5].

To help clinicians adhere to evidence-based guidelines and deliver standardized care based on the best-available scientific information, medical algorithms have been developed, such as in the treatment of hypertension, diabetes, high cholesterol, cancer and myocardial infarction. Similarly, in psychiatry the Texas Medication Algorithm Project developed an algorithm to assess its value in managing the pharmacological treatment of patients with schizophrenia, bipolar disorder and major depressive disorder [6]. Even though clinical outcomes of patients whose psychiatrists used this algorithm reported a statistically significant benefit, studies have consistently shown that the initial benefits of algorithm implementation are not sustained once the implementation support is withdrawn [7].

Moreover, for the algorithm to truly become a clinical tool, innovative methodologies are needed to support probabilistic decision-making and incorporate patients’ views and clinical judgement in a dynamic way [8]. A difference in efficacy between interventions of 5%, for instance, might mean more to one patient than to another and it does not precisely exclude a benefit that clinicians and patients might find meaningful. Or, vice versa, the same result could allow some doctors and patients to choose to avoid the treatment after careful consideration of tolerability, risk and uncertainty.

For this reason, we have developed the prototype of a new, cloud-based clinical decision support tool (CDST), a treatment algorithm that aims at providing updated and stratified information about interventions for different subgroups of patients. To test the acceptability of the CDST, we designed a focus group study that aimed to explore clinicians’, patients’ and carers’ perspectives on the algorithm prototype. In particular, we wanted to explore whether participants felt it was a useful decision-making tool for improving prescribing practices in routine psychiatric care.

## Methods

**Fig. 1:**
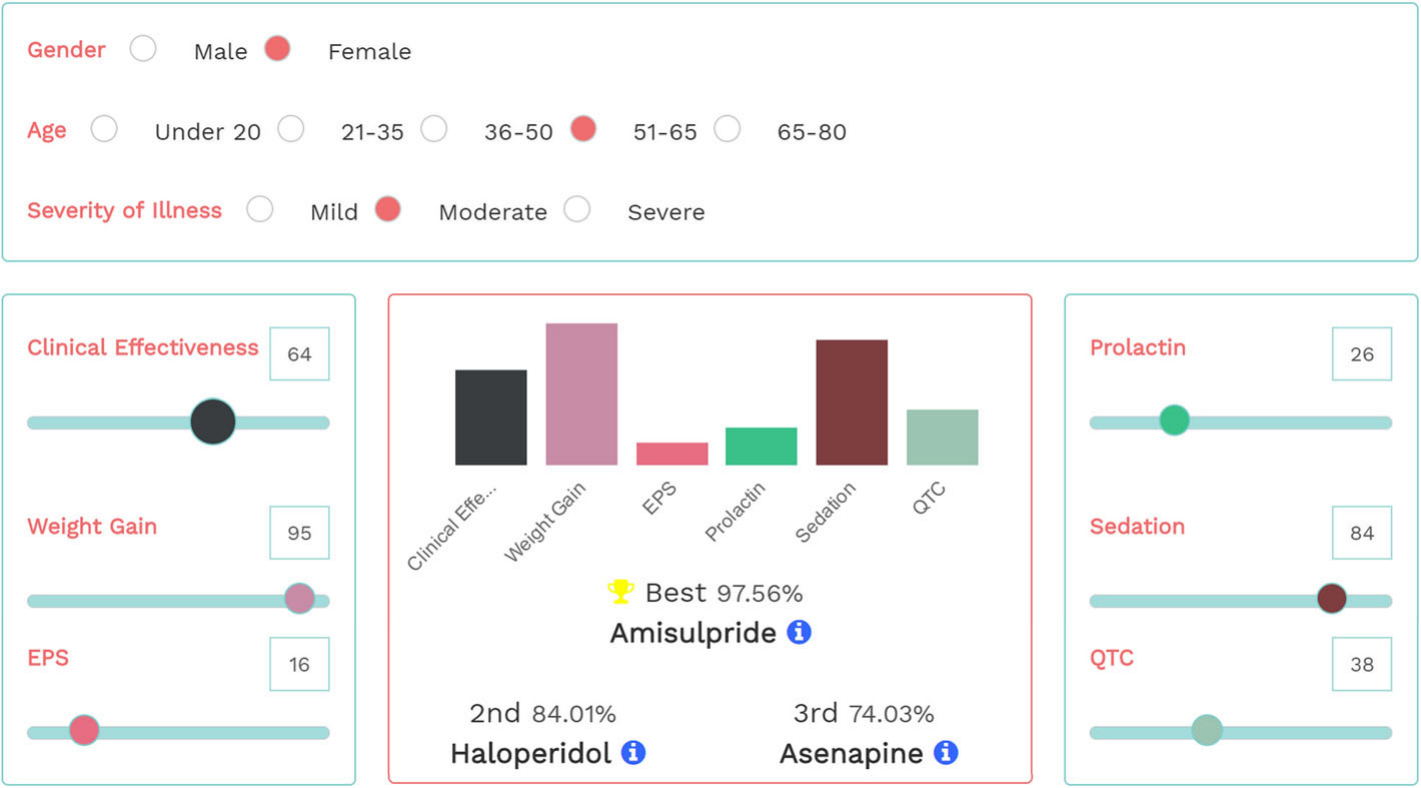
Layout of the clinical decision support tool (CDST). The CDST allows clinicians and patients to enter simple demographic and clinical variables (i.e. age, gender, severity – *top box*) and discuss the relevance of the different side effects (there is a score 0 to 100 to select the best tolerability profile according to personal preferences – *left and right boxes*). At the centre of the figure, results are presented as *bar graphs* reporting the percentage allocated to each side effect and the ranking of the treatments, with the corresponding probability to be the best, the second best, etc. In this working example, the patient is a female, aged between 51 and 65 years old, with moderate severity of symptoms, who is really concerned about weight gain and sedation (the higher the score, the more important the adverse event to avoid). Legend: EPS, extra-pyramidal symptoms; QTc, corrected QT interval

The CDST was developed by researchers at the University of Oxford in collaboration with experts working at the University of Tel Aviv (Fig. 1) and is part of a larger research programme, which proposes to develop an integrated system using remote technology to monitor clinical outcomes using a machine learning approach. To tailor treatments at an individual level, the CDST balances stratified recommendations from network meta-analysis (the best methodological design for comparative effectiveness) [9] in combination with the preferences and values of patients, carers and clinicians. To incorporate individual preferences, we used bar graphs reporting the percentage allocated to each side effects and the ranking of the treatments based on the results from the network meta-analysis. The final ranking of the best five interventions will depend not only on the clinical and demographic characteristics of the patient (information derived from the randomised data) but also on the individual choice in terms of tolerability profile (subjective preference). As a working example for the CDST, we decided to use data from randomised trials about efficacy and acceptability of pharmacological treatments in schizophrenia [10]. However, the focus group discussion focused on the layout and applicability of the CDST, rather than the clinical content of the information provided.

We chose to use focus groups to investigate acceptability, to encourage participants to openly explore their own and others’ perspectives collectively, with opportunity for clarification and debate, without using a rigid framework. Three separate focus groups were planned: one for consultant psychiatrists working in the National Health Service (NHS) in the UK, one for patients and carers, and one for general practitioners and nurses. We conducted separate focus groups to allow participants to speak freely without feeling inhibited in the presence of other group members, thus facilitating discussion.

We checked with the local Ethics Committee and ethical approvals were not required for the study, however written informed consent was taken from each participant.

### Access, recruitment and participant characteristics

The study took place in Oxford (United Kingdom) between October 2016 and January 2017. We conducted three focus groups comprising of three different participant types. The first was for Consultant Psychiatrists working in the Oxford Health NHS Foundation Trust each of which received an invitation and participant information sheet via email. The second was for participants who had a prior mental health diagnosis and/or experience of caring for someone with a mental health diagnosis. They received a modified version of the participant information sheet in Plain English to ensure understanding. We managed to have a diverse representation of mental health disorders, including schizophrenia, major depression, bipolar disorder, anorexia and anxiety. Participants for this group were initially identified using a local Patient and the Public Involvement and Engagement (PPI/E) group, followed by purposive sampling to ensure diversity in age, gender, patient and carer experience and technological abilities. The third focus group comprised of primary care general practitioners and nurses, following invitation to participate from a local general practitioner (GP) practice.

Overall 31 participants attended the focus groups. Of these 12 were consultant psychiatrists, 11 primary care practitioners (general practitioners *n* = 6; nurses *n* = 5) and eight were patients or carers. The majority of participants were white British (*n* = 24, 77.4%), women (*n* = 17, 54.8%) and aged over 45 years (*n* = 19, 61.3%) (Table 1).

**Table 1:** Characteristics of focus group participants

Participant demographics		Psychiatrists (*N* = 12)	General practitioners (*N* = 11)	Patients and carers (*N* = 8)	Total (*N* = 31)
Age (years)	30–45	4	7	1	12
	> 45–60	7	4	2	13
	> 60	1	0	5	6
Gender	Male	6	3	5	13
	Female	6	8	3	17
Ethnicity	White (British)	7	9	8	24
	White (Other)	5	1	0	6
	Afro-Caribbean	0	1	0	1

Both in the group of consultant psychiatrists and in the group of general practitioners there were clinicians who had been practicing for a long time (more than 20 years) as well as clinicians who were less experienced (fewer than 10 years).

Each focus group lasted around one hour and started with a short, interactive, visual demonstration of how the CDST worked, before answering any questions relating to the demonstration. To standardise the procedure, we used the same topic guide that covered themes relating to the acceptability and usability of the CDST (Table 2).

**Table 2:** Topic guide used in focus group study

Questions	
• Can you summarise what you think the clinical decision support tool will be used for?	
• Can you tell me what you think about the layout of the clinical decision support tool?	
• What are the strengths and weaknesses of using this tool in clinical practice?	
• Can you tell me what you like and don’t like about this clinical decision support tool?	
• Can you think of anything that could be added or removed from the clinical decision making tool to improve it?	
• How do you think patients and carers will respond to the clinical decision support tool? Do you think it will impact on the doctor-patient relationship? If so, how?	
• Are there any patient groups for whom this tool may be particularly useful or unhelpful?	
• Are there any advantages or disadvantages of using this tool in clinical practice?	
• What do you think about the web-based interface?	
• How do you feel using an electronic tool compares to using more traditional methods in clinics?	

### Data analysis

**Fig. 2:**
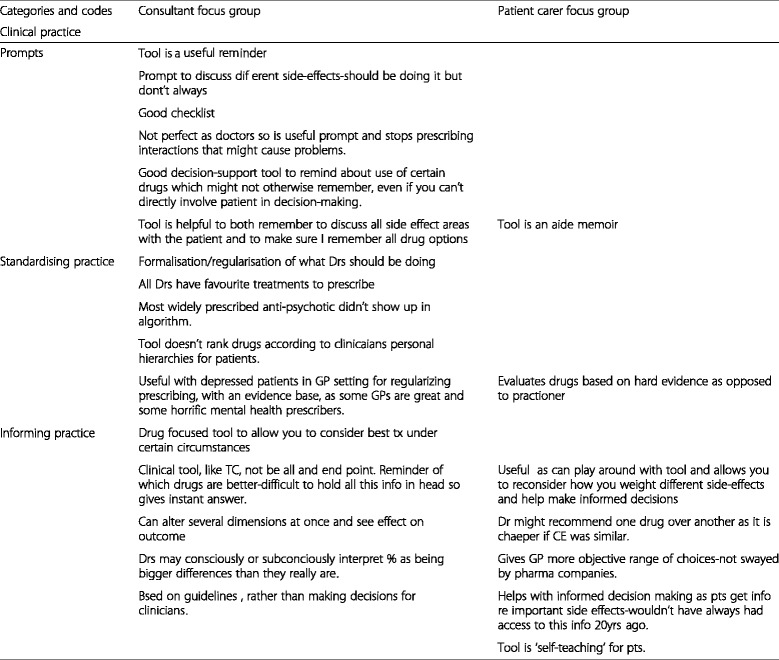
Snapshot of the Framework Matrix used to undertake qualitative data analysis process

Focus groups were recorded using a digital voice recorder before being transcribed by a local transcription service. Any identifying participant details were anonymised and the focus group recordings were then removed from the digital voice recorder and transferred to a secure, password protected storage facility in line with the universities’ policies on data storage and protection. Data were analysed thematically and managed using the Framework method [11]. Transcripts were coded and a working analytical framework was established. Using the constant comparative method [12], any similarities and differences in perspectives between clinicians and patients/carers were established with regard to the CDST. Transcript data were manually inserted into a Framework matrix (Fig 2) to enable ordering and data synthesis [11]. This enabled within and across case analysis of the data from the three focus groups identifying key themes relating to participants’ views on the CDST and its application in the clinical setting.

## Results

The main themes that emerged related to CDST *applications in clinical practice*, *communication*, *conflicting priorities*, *record keeping and data management*. Table 3 presents a summary of the main themes reported on.

**Table 3:** Summary of main themes emerging from the focus group dataset

Themes	Applications in clinical practice	Communication	Conflicting priorities	Record keeping and data management
Key points raised	• Clinical applications in psychiatry and other chronic conditions • Supports standardized decision making for prescribing • Reminder to consider all treatment options • Prompts discussion between clinicians and patients • Boosts prescribing confidence • Useful learning tool • Promotes informed choice • Enhances, but not replacement for, clinical judgment • Does not reflect clinical assessment process	• Promotes discussion about medication side-effects • May cause information overload • Facilitates informed consent • Treatment choice of patient and clinician may be at odds • May optimise capacity, by teasing out views • Level of collaboration may vary • Terminology too “medicalised” • Improved presentation will facilitate discussion • Promotes collaboration but may cause uncertainty in decision-making • May increase compliance	• Patient’s healthcare priorities need identifying to ensure clinicians consider them • Promotes personalised care; but tool too disease-focused. • Potential conflict if patients feel they are being denied recommended treatments; only available medications should be used in CDST • Full range of CDST recommendations could be used to challenge clinical commissioners	• Good record of consultation • Potential to link CDST to patient records • Continually updated evidence-base • Outcomes presented are understandable • Concerns about data reliability • Easy to use • Time saving: hard to use within 10 min consultation, but pays off long term

Whilst in general, there was more congruence than divergence in the perspectives of the consultant, primary care and patient and carer focus groups, the views of the groups were sometimes nuanced, based on their contextual needs, values, priorities and concerns. For example, whilst all three groups viewed the CDST as a useful and collaborative learning and decision-making tool, the consultant group expressed concern that it should not be viewed as a replacement for clinical judgement, whilst a prominent concern of Primary Care participants was that patients might become overloaded with worry if provided with too much information. However, the patients and carer group felt the amount of trust they had in their doctor would be a key factor in the shared decision making process. The study findings will now be reported on in more detail.

### Applications in clinical practice

#### Positive comments

All participants agreed that the CDST has the potential for clinical applications in psychiatry. The majority reported that it was a useful tool to support decision making for prescribing. They felt the tool reminded clinicians to consider all available treatment options (not just the most effective, well known, or best tolerated). They thought it prompted discussion between clinicians and patients around potential side-effects, best evidence and drug interactions: “*I think it is a useful tool because we’re not perfect so we will forget things and we will also prescribe drugs with interactions that are going to be a real problem.” (Consultant 12).*

Most consultants viewed the CDST as an effective way to standardise prescribing practices, by ranking interventions according to their comparative efficacy and side-effect profiles. This was seen as a more favourable method than relying on personally derived drug hierarchies, with some consultants admitting that their prescribing preferences were not always based on the most up-to-date evidence: “*For me it almost seems like a formalisation or a regularisation of what should be good practice anyway with the information in there.” (Consultant 12).*

The tool was perceived to be useful by clinicians who had been practicing for a long time as well as clinicians who were relatively new to practice. All clinicians reported the CDST would be useful to help remember and retain information about the severity of different treatment side-effects across the different interventions, especially when there is little difference between certain drugs in terms of efficacy, boosting their prescribing confidence as a result: “*This is… a way of quickly reminding us…which [drugs] are better…I always hold in my head actually which one is the best for [patients with] QTc prolongation … It gives you an instant answer.” (Consultant 1).*

Primary care participants suggested that the CDST could inform prescribing practice by providing a history of patients’ previous side-effects. They also felt that the CDST provided a useful learning tool for clinicians if they selected a lower ranking drug than the recommended CDST treatment, by prompting them to question their rationale for doing so: “*You've asked the patient what's important to them…It's come up with that outcome based on that…It's a really useful learning tool for us to be thinking about what our prescribing choices are” (Primary Care 5).*

Patients and carers described the CDST as ‘self-teaching’, helping them make informed choices and allowing them to readily access information than they previously did not have: “*It helps in making informed decisions because 20 years ago people weren’t being told about side effects necessarily so they couldn’t even make important choices.” (Patient and Carer 7).*

#### Suggestions for change or improvement

Some consultants were wary of the CDST being used as a replacement for clinical judgement and expressed concern about “becoming blinkered by it”. They reported the tool did not reflect the complex contextual clinical assessment process, being unable to capture detailed information about patient characteristics, time pressures, anxiety, influence of carers, clinician experience and organisational factors. Some clinicians said that this provided a significant limitation to the CDST’s usefulness: “*When we see someone… it changes completely the context of the conversation…Depending on how much time you have or depending on whether the person is having an attack or not which I did not capture in this [tool].” (Consultant 9).*

However, other clinicians thought that the CDST informed and enhanced clinical judgement, by providing unbiased, credible, evidence-based information, thus removing some of the reticence about prescribing drugs that were not routinely used in practice: “*Having this kind of information…Would make you more likely to go and look at the evidence for the drug that's being recommended if it's one that you're not used to.” (Primary Care 9).*

For patients and carers the CDST encouraged them to consider different factors during the clinical assessment process, rather than just focusing on one or two things: “*That’s what I like about the approach, it does assist judgement, you’re not committing to anything, you’re comparing bits.” (Patient and Carer 3).*

All participants agreed that the CDST could be widely used for other chronic conditions where medications have to be tailored and adjusted over time such as other psychiatric conditions (eg depression, diabetes, asthma and hypertension).

### Communication

#### Discussion of potential benefits and challenges

One major benefit of the CDST was perceived to be that it opened up discussion about the possible side-effects of medications. Some clinicians commented that the CDST provided opportunity to fully discuss medications’ tolerability profile with patients, which they did not otherwise always do. This was important, as clinicians thought that the way they convey the severity of side-effects to patients is likely to influence their subsequent drug preferences: “*How you explain some of these things to patients [will]…very much influence what weight they give to them.” (Consultant 8).*

However, some primary care providers and patients/carers highlighted that there are disadvantages to receiving too much information about possible medication side-effects, as it might cause unnecessary worry and result in clinicians spending considerable time reassuring patients. Whilst it was important they were made aware of the main side-effects from treatment, the majority of patients and carers did not feel they needed to know every detail, so long as their doctor was well informed. This view was echoed by some consultants who reported there was a danger of entering into a *‘minefield of discussion’*.

Most clinicians said that the CDST could help facilitate the informed consent process, though a few thought it might cause problems for patients with limited intellectual capacity, creating tensions if their preferred treatment choice was at odds with their clinician’s. However, generally it was felt that the CDST could optimise capacity, by teasing out patients’ views and preferences, though the degree of clinician/patient collaboration might vary: “*Even somebody who’s quite psychotic might have a fairly good idea of what side effect profile they might prefer.” (Consultant 5).*

#### Suggestions for change

Participants from all the three groups universally commented that the terminology and language presented on the CDST were too “medicalised”, making it difficult for lay people to understand and potentially lead to ethical issues around consent to treatment, as patients. There was consensus that simpler language was required and that clinicians needed a pre-determined knowledge of the demographic and level of understanding of patients, to avoid having to provide a *‘series of tutorials’* to explain every acronym: “*What concerns me is that it’s very easy for a clinician to lead you at that point if you don’t really understand what it is you’re looking at.” (Patient and Carer 4).*

Each group made recommendations for improving the way the information was presented (including relabelling and resizing scales, adding pictorial representations of side-effects or increasing interactivity). This would facilitate discussions with patients and raise their awareness of different side-effect profiles: “*The interface needs to be simplified and have lay terms and more explanation…To facilitate the conversation a bit more.” (Primary Care2).*

Showing the CDST’s screen to patients during a consultation was a matter of discussion. Many patients said that it could be distressing, causing confusion and uncertainty during the decision-making process. However, some patients and carers felt the CDST should be patient-facing to promote collaboration in decision-making, so long as this did not impinge on their clinician’s duty of care: “*The information must be there because if we’re to be transparent and it’s to be a co-production…To build trust and so forth. The patient should have access to the same information provided it doesn’t override the doctor’s duty of care to that patient.” (Patient and Carer 8).*

A couple of carers thought that having access to the CDST interface might lead to some patients manipulating the tool until they received their preferred drug of choice. However, primary care practitioners reported that patients would require a lot of knowledge for this to happen: “*It would take quite a lot of knowledge to know which ones to move…It up the top.” (Primary Care 11).*

One patient commented that some clinicians may not respond well to patients having an increased level of control over the decision-making process, whilst others felt that trust in the clinician-patient relationship was vital for ensuring successful decision-making, something the CDST could not control for: “*A doctor is not going to respond…well to somebody having done this and then say, actually, what I need is…”. (Patient and Carer 1).*

Primary care practitioners commented that the CDST could increase patient compliance if it was tailored to their needs: “*If you’re helping people to tailor the things that they prefer to avoid, I would hope that that would improve compliance.*” *(Primary Care 9).*

### Conflicting priorities

Clinicians, patients and carers reported that prior to using the CDST patients should be encouraged to declare a checklist of their healthcare priorities, to assist with shared decision-making, ensuring mismatched priorities are identified and that their needs, views and perspectives are listened to. This was viewed as a valuable way of ensuring clinicians did not purely rely on their past prescribing preferences: “*What the patients come up with…They’ve said what they do or don’t want…Use that as a guide of what we should be thinking of using.*” *(Primary Care 5).*

Many clinicians, patients and carers agreed that the CDST promoted personalised care. However, they recognised that the CDST was at times too disease-focused and could not compensate for the lifestyle and human factors that influence decision-making. The possibility of incorporating other data into the CDST such as genes and demographics was raised, to increase the CDST’s accuracy: “*What would make [the tool] potent is if you could start to include more biological data about patients.” (Consultant 4).*

Other conflicting priorities were highlighted by primary care practitioners who suggested that increasing pressures on prescribing budgets meant the CDST should only identify medications available from within their own clinical commissioning groups. They commented that conflict could arise if patients felt they were being denied access to recommended CDST treatments which were not commissioned in their local area: “*The cost effectiveness and the limited choice is very important…If something flashes up, potentially it can lead to some problems later down in the relationship that they're not getting the best thing.” (Primary Care 2).*

However, from an educational perspective, it was thought helpful to have access to the full range of CDST recommendations, regardless of whether they were available from their clinical commissioning group. This could allow the commissioners to be challenged about why certain medications were not made available, as well as facilitating data collection to compare local prescribing practices against CDST recommendations: “*From the education point of view, then more information about more drugs is fine. Because you can go away and talk to your clinical commissioning group about why this one isn't available.” (Primary Care 9).*

### Record keeping and data management

According to the majority of clinicians, patients and carers, the CDST could provide a good record of the consultation. However, suggested improvements included linking the CDST to patient clinical records’ database so that individuals’ medication histories could be incorporated. Participants praised the CDST’s ability to continually update its evidence-base, build on existing best available literature and present these outcomes in a quantitative and understandable way. However, some consultants expressed concern about the reliability of data from the network meta-analysis, commenting on the lack of external validity of randomised controlled trials: “*It’s a highly selected group of patients ….” (Consultant 4).*

Finally, most participants thought that the CDST was easy to use, encouraging clinicians to use it on a daily basis. The CDST was commended for focusing on one specific aspect of the clinical consultation, saving time by collating relevant drug information within one database: “*This particular tool serves a specific purpose…It’s about deciding on how to treat them pharmaceutically….I think it’s fantastic.” (Patient and Carer 6).*

A few primary care practitioners said that it could be hard to use the CDST within a 10 min consultation period, however they argued that this initial time investment would pay off long term: “*If it comes to enabling people to make a better choice, that's quite a major time saving rather than having them coming back saying, oh I didn't like it.” (Primary Care 11).*

## Discussion

The findings from this study provide useful insights into the perspectives of clinicians, patients and carers with regard to the usefulness of clinical support tools. They have led the research team to consider how the CDST might be optimised in the clinical setting to enable it to be mutually beneficial to clinicians and patients. The majority of participants felt the CDST could play a role in regulating and standardising prescribing processes in routine mental health practice.

Previous research has shown that computer assisted guidance on drug prescribing can improve patients’ therapeutic outcomes, reduce costs and result in more effective care outcomes [13]. This is something that participants found the CDST valuable for, allowing them to base their decision-making on verifiable, up-to-date evidence and challenging their own assumptions about best prescribing practice. The CDST has the potential to build on good prescribing practice by weighting the clinical effectiveness of drugs against the severity of side-effects. This will enable clinicians, in conjunction with patients, to make appropriate medication choices at an individual level. Although some participants commented that the CDST was too medicalised, this referred at large to the terminology and language used. The CDST’s concept, although based in biomedicine, engages with a holistic approach to care and is embedded in the principles of personalised medicine and informed choice. This concept of informed choice is critical in a society where patients increasingly seek engagement in their healthcare choices, to ensure that they have a better understanding of the nature of their illness, against the backdrop of an increasingly complex and fragmented healthcare system [14, 15]. The CDST can help enable patients to make the right healthcare decisions for themselves within the context of their own lives, even if this maybe at odds with the views and perspectives of clinicians [14].

As the findings highlighted, the CDST is not intended to be a replacement for clinical judgement. The complexities surrounding the clinical assessment, management and treatment of mental health patients is multi-factorial. It depends on human, environmental, cultural, social, practical, physical and psychological factors and will not always fit neatly within the medical model of care or the CDST parameters. Although some clinicians expressed concern that the CDST may blinker doctors and stop them from considering other factors, we argue against this. Just as clinicians rely on summaries about patients’ social circumstances, comorbidities and test results to help build a comprehensive history, this background information does not inhibit them from listening and responding to the immediate needs of patients. Similarly, having access to evidence-based medication recommendations should not inhibit clinicians from employing their own clinical judgement. Rather, these recommendations form one piece of a complex jigsaw that requires knowledge, experience and expertise to complete [16].

The findings revealed that clinicians, patients and carers placed value in the CDST’s ability to facilitate discussion about the side-effect profiles of different psychiatric drugs. A study carried out to ascertain whether patients knew their diagnoses, treatment plans and common side effects of prescribed medications, found that less than half were able to list their diagnoses, medication names and purpose, or associated medication side-effects [17]. This suggests an apparent lack of communication between clinicians and patients. The CDST can help rectify this, by pinpointing areas for discussion that clinicians might otherwise omit and by drawing specific side-effects to the attention of patients. The time saving elements of the CDST may also facilitate improved communication pathways between patients and clinicians, by focusing on priority areas for discussion. However, care must be taken to consider the level of information desired by individual patients to avoid causing distress, confusion or pressure in decision-making.

The issue of informed consent and capacity is integral to decision-making in mental health. Research has shown that deficits in understanding among individuals with psychiatric illness or cognitive impairment can be improved with educational interventions, especially when they are presented in an organised and simplified manner [18]. Furthermore, multimedia resources which are interactive, incorporate visual aids, are simple to use and have a clear focus, have been shown to help align information to individual needs and promote active engagement from participants [19]. This highlights the need for modifications to be made to the CDST, to ensure that the language and design features are easy to understand and engage with, especially for mental health patients who may have limited capacity. In addition, medications appearing on the CDSTs interface could be stratified to ensure they align with those available within specific healthcare localities.

The main limitation of this study is that patients vary substantially in how much they wish to contribute to the decision-making process [20]. Whilst some of them with little input may be content with this, trusting their clinician to make a treatment decision using the best available evidence, other patients may view this as paternalistic, remiss and lacking in understanding of what their personal health priorities might be. Collaborative decision-making between clinicians and patients can empower patients, with positive impacts on their subsequent health outcomes [14, 15, 21]. The CDST has been designed to have a shared interface between clinicians and patients, however the level of collaboration is subjective and dependent on the level of trust and reciprocity within the clinician-patient relationship. Good clinical judgement, an awareness of contextual factors and good communication skills must be employed by clinicians to succinctly assess the level of collaboration required on an individual basis, support shared and collaborative decision making and hence therapeutic relationships and good clinical outcome [22].

Finally, the CDST was generally considered to be an efficient, time-saving clinical tool and this is important given escalating pressures on healthcare resources making clinicians and patients aware of the brevity on their consultations [23]. To this respect, it is worth noting that research suggests that the quality, rather than the length, of the consultation has the biggest impact on patients’ satisfaction [24]. The CDST can support clinicians to navigate patient treatment and management plans, whilst providing assurances that they are utilising a robust and up to date evidence base, with regards to prescribing practices. The study findings will guide future development and evaluation of the CDST to ensure that any modifications made reflect the preferences, needs and priorities of clinicians and patients. Further testing of the algorithm based CDST is planned to ensure that the evidence used to inform it is both credible and reliable.

## Recommendations

The CDST in the mental health and primary care settings can be a useful clinical decision support, with recognised value in promoting clinician-patient collaboration and contributing to the development of personalised medicine. The CDST can improve communication pathways between patients and clinicians, identifying care priorities and providing an up-to-date platform for implementing evidence-based practice. To further this aim, work is planned with PPI/E representatives to ensure that the medical terminology on the CDST is simplified to make it accessible to lay populations. Whilst the CDST has been designed to focus on prescribing practices within a mental health context, other evidence-based algorithms could be designed to incorporate factors such as demographic variations and non-pharmacological interventions, as well as a variety of mental and physical health disorders. Primary care practitioners may value the CDST for managing and treating patients with long term conditions such as hypertension and diabetes, within short clinic time slots. Primary care practitioners require a broader range of knowledge than practitioners in more specialised secondary and tertiary care services. The CDST may be particularly valuable in these circumstances, by providing up-to-date, evidence based information on a variety of health conditions in a timely manner, something that is essential given the time-limited consultations available.

## Conclusions

As well as providing a tool to support evidence-based practice, we plan to use the CDST to gather feedback from patients to increase its precision, by effectively incorporating everyday clinical information from ‘real-world’ individuals with the best available evidence that is contained within the CDST. This has the potential not only to improve treatment outcomes, but also to strengthen the evidence base around the interaction of specific drugs with other pharmacological and psychosocial interventions, whilst accounting for individual characteristics and patient preferences.

Future research will also involve a pilot randomised controlled trial to test the CDST. If shown to be implementable in routine clinical practice in different settings, the CDST could lead to changes in prescribing practices of clinicians, protecting patient safety, whilst paving the way for precision medicine in every-day mental health care.

## Abbreviations

CDST: Clinical decision support tool
GP: General practitioner
PPI/E: Patient and the public involvement and engagement
QTc: Corrected QT interval
